# Data in support for the measurement of heparan sulfate and dermatan sulfate by LC–MS/MS analysis

**DOI:** 10.1016/j.dib.2018.11.100

**Published:** 2018-11-26

**Authors:** Giulia Forni, Sabrina Malvagia, Silvia Funghini, Emanuela Scolamiero, Massimo Mura, Maria Della Bona, Fabio Villanelli, Roberta Damiano, Giancarlo la Marca

**Affiliations:** a Newborn Screening, Biochemistry and Pharmacology Laboratory, Pediatric Neurology Unit and Laboratories, Meyer Children׳s University Hospital, Florence, Italy; b Department of Experimental and Clinical Biomedical Sciences, University of Florence, Florence, Italy

## Abstract

This article provides supplementary data for the paper “LC–MS/MS method for simultaneous quantification of heparan sulfate and dermatan sulfate in urine by butanolysis derivatization” (Forni et al., 2018). Several parameters were tested to optimize sample preparation by butanolysis in order to carry out simultaneous quantifications of HS and DS by tandem mass spectrometry.

Here we describe step-by-step instructions to perform HS and DS analysis in urine samples using external calibration curves of standards of known concentration. Sample are quantified by interpolation from the calibration curve and reported in µg/mL. Then, HS and DS are normalized to creatinine concentration and reported as mg/g uCr.

**Specifications table**

**Table:** 

Subject area	Clinical Chemistry
More specific subject area	Heparan and Dermatan sulfates quantification in urine samples.
Type of data	Figures
How data was acquired	Chromatographic separation of derivatized urine samples using an Agilent 1260 Infinity HPLC capillary system with Kinetex Biphenyl column 2.6 µm, 100 × 2.1 mm (Phenomenex, Torrance, CA) coupled to a AB Sciex QTRAP 5500 for MS/MS analysis.
Data format	Filtered and analyzed.
Experimental factors	Derivatization of urine samples by butanolysis followed by LC–MS/MS analysis for simultaneous quantification of heparan and dermatan sulfates.
Experimental features	Optimization of butanolysis reaction conditions for deriving HS and DS.
	Standard operating procedure for sample preparation.
	Stability study.
Data source location	Florence, Italy.
Data accessibility	Data is with this article.
Related research article	Forni G, Malvagia S, Funghini S, Scolamiero E, Mura M, Della Bona M, Villanelli F, Damiano R, la Marca G. LC–MS/MS method for simultaneous quantification of heparan sulfate and dermatan sulfate in urine by butanolysis derivatization. Clin. Chim. Acta 488 (2018) 98–103 [1].

**Value of the data**

• The described method allows the simultaneous quantitation of dermatan sulfate (DS) and heparan sulfate (HS) in urine by LC–MS/MS, so as to facilitate differential diagnoses in MPS and targeted patient follow up.
• The protocol for preparing samples involves the chemical cleavage of glycosaminoglycans (GAGs) in a butanolysis reaction. The high yield product permits a reproducible quantitation of HS and DS even if a small amount of sample is used.
• The method could become a useful and reliable test in clinical laboratories where mass spectrometry is commonly used in several areas of diagnostics.
• Given that the method can be applied to different type of matrices including dried blood spots, it could be modified and adapted for second tier testing of positive samples in newborn screening programmes for lysosomal storage disorders.

## 1 Data

The reported dataset includes five figures.

**Fig. 1:**
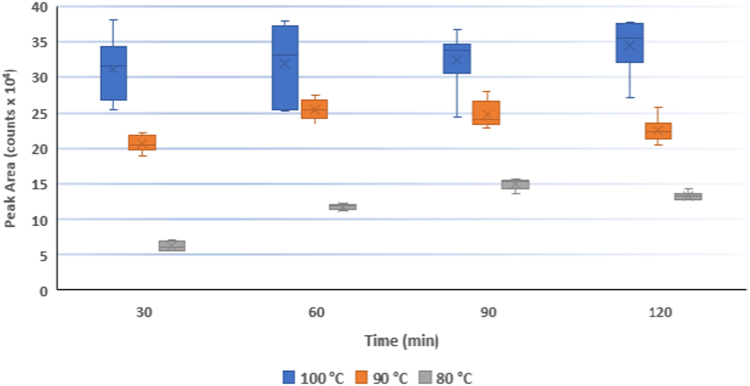
The *y*-axis represents the HS peak area resulting from three different incubation conditions. Boxes represent interquartile ranges, and the horizontal line across each box indicates the median values. The “x” markers represent mean values.

**Fig. 2:**
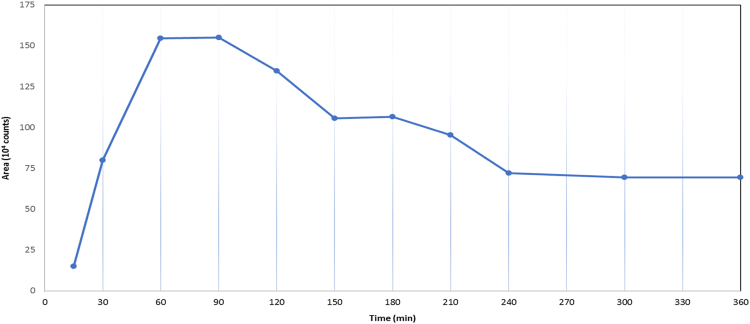
Time lapse of HS butanolysis reaction over a period of 6 h at 90 °C.

**Fig. 3:**
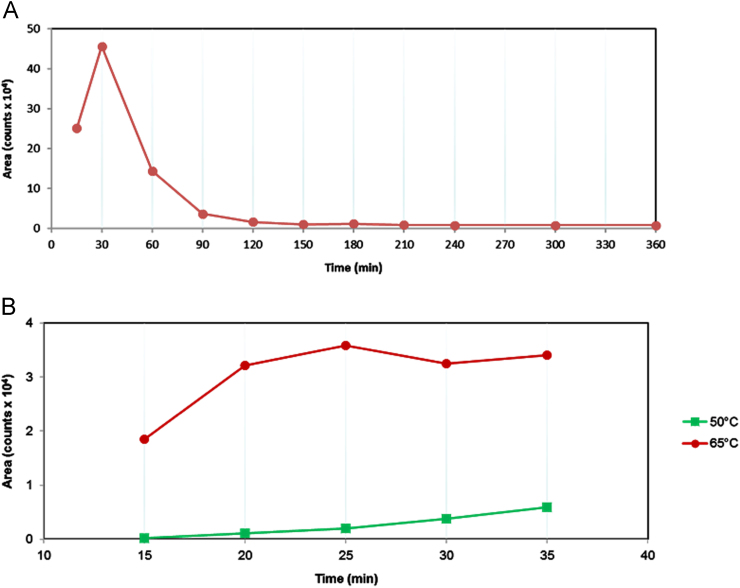
Optimization of DS butanolysis reaction conditions. Signal monitoring was performed during butanolysis over a period of 6 h at 65 °C, (panel A). A shorter time investigation was tested at two different temperatures, 50 °C (blue line) and 65 °C (red line), (panel B).

**Fig. 4:**
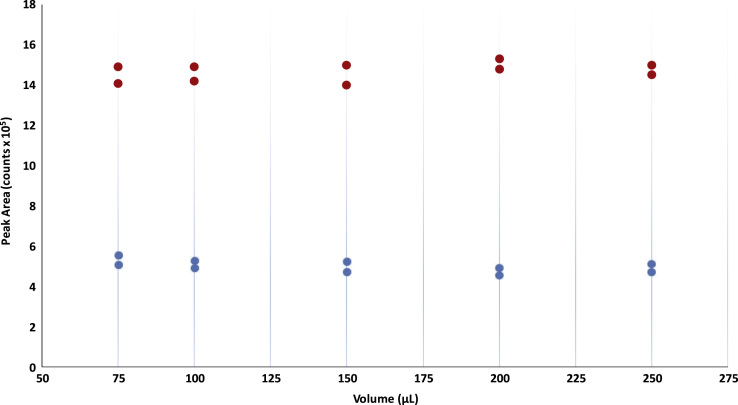
The HS high-QC sample was incubated at 90 °C for 1 h with 75, 100, 150, 200 and 250 µL of 3 N HCl in n-Butanol (red dots). The DS high-QC sample was incubated at 60 °C for 25 min with the same volumes of 3 N HCl in n-Butanol used for HS (blue dots).

Fig. 1, Fig. 2, Fig. 3, Fig. 4 provide experimental data for the optimization of time-temperature and reagent volume parameters for butanolysis reaction of HS and DS.

**Fig. 5:**
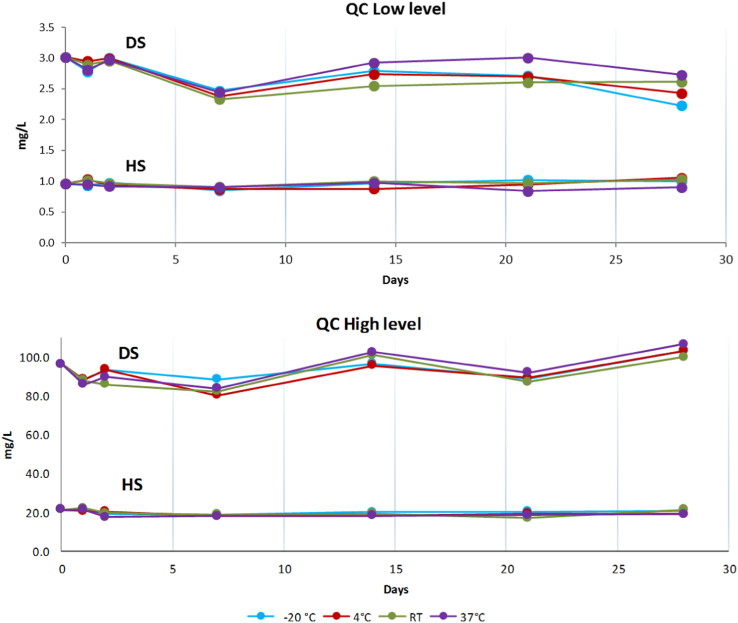
Effect of different temperature storage (−20, 4, 37 °C and room temperature) on stability of DS and HS in urine.

Fig. 5 provides short-term stability data relating to storage conditions.

The details on the operating procedure for the quantitative analysis of HS and DS in human urine are given below.

## 2 Experimental design, materials and methods

### 2.1 Operating procedure for sample preparation

1. Keep the urine sample at room temperature until completely thawed. Gently invert the tube to ensure that the urine specimen is homogeneous.
2. Filter ~1–2 mL of urine with 0.22-µm syringe filters (Merck KGaA, Darmstadt, Germany) and transfer to 1.5 mL tube.
3. Analyze all urine samples for creatinine in order to measure an equal concentration of urine for each patient:
  if the initial creatinine of the sample (i-uCr) is >100 µg/mL:
    a) dilute the filtered urine in deionized water until a final creatinine concentration (f-uCr) of 100 µg/mL is reached:
      i−uCr/f−uCr=dilutionfactor
    b) Label two glass screw-cap tubes for each patient sample, one tube for DS and one tube for HS. Transfer 5 µL of urine (100 µg/L uCr) into each tube to reach an absolute value of creatinine of 500 mg.
  If i-uCr <100 µg/mL:
    a) Use a volume of urine with an absolute value of creatinine of 500 µg
      Thecalculationusedis:500/creatinineconcentration(µg/L)=urinevolume(µL).
    b) Label two glass screw-cap tubes for each patient sample. Transfer the calculated volume of urine into each labelled tube.
4. Dry the samples under a stream of nitrogen at 45 °C.
5. Add 75 µL of 3 N HCl in N-Butanol to each vial working in a fume hood to chemically cleave the glycosaminoglycans. Close the tubes tightly and incubate in a thermostatically controlled oven as follows:
  HS samples at 90 °C for 60 min;
  DS samples at 65 °C for 25 min.
6. Cool the samples to room temperature for 10 min and dry under a stream of nitrogen at 45 °C.
7. Prepare a water/acetonitrile solution (30:70, v/v) containing 0.1% of formic acid. The solution is stable for 1 month at room temperature.
8. Add 200 µL of solution to each HS tube and 800 µL to each DS tube.
9. Vortex for 15 s.
10. Combine DS samples into the correspondingly labeled tube containing HS for each patient (final volume 1 mL)
11. Vortex for 15 s.

Samples can be kept for two days at 4–8 °C.

### 2.2 Calibration standard solutions

12. Prepare two stock solutions for DS and HS at a concentration of 3 and 1 g/L in water, respectively.
13. Prepare a calibration standard stock solution HS7 at 50 mg/L by diluting 1:20 the stock solution at 1 g/L in water. Prepare calibration standard stock solutions from HS6 to HS1 by a serial dilution 1:2 from HS7 calibration standard stock solutions in water.
14. Prepare a calibration standard stock solution DS7 at 200 mg/L by diluting 1:15 the stock solution at 3 g/L in water. Prepare DS6 to DS1 calibration standard stock solutions by a serial dilution 1: 2 from DS7 in water.
15. The calibration standard stocks solutions are as follows:

**Table:** 

**HS calibration standard stock solutions**	**Concentrations (mg/L)**	**DS calibration standard stock solutions**	**Concentrations (mg/L)**
***HS1***	0.78	***DS1***	3.12
***HS2***	1.562	***DS2***	6.25
***HS3***	3.125	***DS3***	12.5
***HS4***	6.25	***DS4***	25
***HS5***	12.5	***DS5***	50
***HS6***	25	***DS6***	100
***HS7***	50	***DS7***	200

### 2.3 Preparation of an external calibration curve

16. Dilute the synthetic urine with water until a final uCr of 200 µg/L is reached.
17. Prepare the calibration standards for HS and DS from 0 to 7 by adding 200 μL of each calibration standard stock solution to 200 μL of synthetic urine (uCr 200 µg/L).
18. Transfer 5 µL of each calibration standard to a labeled tube and proceed to point 4.

Normalize the results value for urinary creatinine and express it as mg/g of uCr.

### 2.4 Preparation of quality control samples

19. Prepare the High and Low QC stock solutions for HS at 40 mg/L and 2 mg/L by diluting 1:25 and 1:500 the stock solution at 1 g/L in water, respectively.
20. Prepare the High QC stock solution for DS at 180 mg/L by adding 90 μL of the stock solution at 3 g/L to 1410 μL of water. Prepare the Low QC stock solution for DS at 6 mg/L by diluting 1:500 the stock solution at 3 g/L in water.
21. Prepare the Low and High QC samples combining the same volume of each QC stock solution with synthetic urine (uCr 200 µg/L) obtaining the following levels:

**Table:** 

Analyte	QC samples	mg/L
HS	Low	1.0
	High	20.0
DS	Low	3.0
	High	90.0

### 2.5 Optimization of sample preparation

In order to determine the optimal reaction conditions for HS derivatization, a set of experimental runs was conducted to assess the effects of temperature, time and derivatization reagent volume on yield.

Taking into account the results published by Trim [2], we tested temperatures around 100 °C. Three sets of spiked samples with fixed HS concentration were incubated at 100, 90 and 80 °C and analyzed every 30 min. Each experiment was performed in six replicates to estimate the variability of results.

The boxplot in Fig. 1 shows the median, interquartile range, and outliers for each dataset. A further dataset was collected to determine the effect of incubation time on the HS peak area counts over a period of 6 h at 90 °C (Fig. 2).

To establish the optimum reaction parameters for DS butanolysis the same experimental procedure used for HS analysis was conducted.

In order to check if different volumes of derivatization reagent corrupt sensitivity, a set of samples for each analyte was incubated with increased amounts of 3 N HCl in n-Butanol (Fig. 4)

QC samples with low and high concentrations were maintained at four different temperatures for 1 month and analyzed at regular time intervals (Fig. 5).
