# Supplementary material for: Data in support for the measurement of heparan sulfate and dermatan sulfate by LC–MS/MS analysis
Source: Data Brief. 2018 Nov 26;21:2398–404. doi: 10.1016/j.dib.2018.11.100 (PMC6282625; doi:10.1016/j.dib.2018.11.100)
Supplement: Supplementary file 1 — Supplementary material. [file mmc1.pdf]

Università degli Studi di Firenze

Florence, 16 November 2018

We wish to confirm that there are no known conflicts of interest associated with this publication and there has been no significant financial support for this work that could have influenced its outcome.

We confirm that the manuscript has been read and approved by all named authors and that there are no other persons who satisfied the criteria for authorship but are not listed. We further confirm that the order of authors listed in the manuscript has been approved by all of us.

We confirm that we have given due consideration to the protection of intellectual property associated with this work and that there are no impediments to publication, including the timing of publication, with respect to intellectual property. In so doing we confirm that we have followed the regulations of our institutions concerning intellectual property.

We understand that the Corresponding Author is the sole contact for the Editorial process (including Editorial Manager and direct communications with the office). He/she is responsible for communicating with the other authors about progress, submissions of revisions and final approval of proofs. We confirm that we have provided a current, correct email address which is accessible by the Corresponding Author and which has been configured to accept email from g.lamarca@meyer.it

LIST AUTHORS

Giulia Forni 16/11/2018

Sabrina Malvagia 16/11/2018

Silvia Funghini 16/11/2018

Emanuela Scolamiero 16/11/2018

Massimo Mura 16/11/2018

Maria Della Bona 16/11/2018

Fabio Villanelli 16/11/2018

Roberta Damiano 16/11/2018

Giancarlo la Marca 16/11/2018
